# Enhancing the Mechanical and Thermal Properties of Epoxy Resin via Blending with Thermoplastic Polysulfone

**DOI:** 10.3390/polym11030461

**Published:** 2019-03-11

**Authors:** Zeyu Sun, Lei Xu, Zhengguo Chen, Yuhao Wang, Rogers Tusiime, Chao Cheng, Shuai Zhou, Yong Liu, Muhuo Yu, Hui Zhang

**Affiliations:** 1 State Key Laboratory for Modification of Chemical Fibers and Polymer Materials, College of Materials Science and Engineering, Donghua University, Shanghai 201620, China; sunzeyu@dhu.edu.cn (Z.S.); xu1758974477@163.com (L.X.); chenzhengguo1@outlook.com (Z.C.); 161100242@mail.dhu.edu.cn (Y.W.); 317001@mail.dhu.edu.cn (R.T.); chengclove_1991@163.com (C.C.); zhoushuaichn@foxmail.com (S.Z.); liuyong@dhu.edu.cn (Y.L.); yumuhuo@dhu.edu.cn (M.Y.); 2 Shanghai Key Laboratory of Lightweight Structural Composites, Shanghai 201620, China; 3 Center for Civil Aviation Composites, Shanghai 201620, China

**Keywords:** polysulfone, epoxy resin, toughness, reaction induced phase separation

## Abstract

Efficient enhancement of the toughness of epoxy resins has been a bottleneck for expanding their suitability for advanced applications. Here, polysulfone (PSF) was adopted to toughen and modify the epoxy. The influences of PSF on the mechanical and thermal properties of the epoxy resin were systematically studied by optical microscopy, Fourier transform infrared spectrometer (FT-IR), differential scanning calorimetry (DSC), thermogravimetric analyzer (TG), dynamic mechanical thermal analyzer (DMA), mechanical tests and scanning electron microscope (SEM). The dissolution experimental results showed that PSF presents a good compatibility with the epoxy resin and could be well dissolved under controlled conditions. The introduction of PSF was found to promote the curing reaction of the epoxy resin without participating in the curing reaction and changing the curing mechanism as revealed by the FT-IR and DSC studies. The mechanical properties of PSF/epoxy resin blends showed that the fracture toughness and impact strength were significantly improved, which could be attributed to the bicontinuous phase structure of PSF/epoxy blends. Representative phase structures resulted from the reaction induced phase separation process were clearly observed in the PSF/epoxy blends during the curing process of epoxy resin, which presented dispersed particles, bicontinuous and phase inverted structures with the increase of the PSF content. Our work further confirmed that the thermal stability of the PSF/epoxy blends was slightly increased compared to that of the pure epoxy resin, mainly due to the good heat resistance of the PSF component.

## 1. Introduction

Epoxy resins (EP) have been widely used in construction, machinery, aerospace and other related fields due to their low cost, excellent bonding performance, outstanding mechanical properties, easy processability, dimensional stability, superior thermal and chemical resistance [1,2,3]. Unfortunately, the high crosslinking density, large internal stress, poor brittleness and poor impact resistance of the epoxy resins after curing has greatly limited their application in some high-tech fields. Therefore, improving the toughness of epoxy resins has been recognized as a crucial way to expand their applications and has attracted much attention in recent years [3,4,5].

Toughness modification of epoxy resins by rubber elastomer [6,7] is very effective but it reduces the mechanical properties and thermal stability of the epoxy resin. Toughening of the epoxy resin with a small amount of liquid crystal polymer [8,9] achieves a worthy toughness effect and makes the composite material have both the high orientation of the liquid crystal and the 3-dimensional network structure of the epoxy resin. However, thermotropic liquid crystal polymers have a relatively high melting point, thus difficult to process and have poor compatibility with epoxy. When nanoparticles are employed to modify the toughness of epoxy resins [10,11,12], the nanoparticles transfer the external force to the surroundings and induce microcracks in the EP matrix hence achieving purposeful toughening. Nonetheless, the price of nanoparticle materials is generally expensive, and many nanoparticles are prone to agglomeration during the modification process. Thermoplastic materials do not only have good toughness but also possess high modulus and good heat resistance. They therefore would significantly improve the toughness of the epoxy resin when applied as toughening agents. Compared to other methods, involving thermoplastic resins would improve the toughness of the EP without reducing its modulus and heat resistance [12]. Meanwhile, this can also give the epoxy some new properties, such as low water absorption and high thermomechanical performances [13], an area that has become a research hotspot in recent years.

The frequently-used thermoplastics consist of poly(ether-sulfone) (PES) [14,15,16,17], poly(ether-ether-ketone) (PEEK) [18,19], polyetherimide (PEI) [20,21,22], polysulfone (PSF) [23,24,25,26,27] and so forth. Due to a similar molecular structure, PSF has good compatibility with bisphenol A type epoxy resin. Moreover, many studies have shown that PSF modified epoxy resin can achieve a good toughness effect without reduction in the thermal stability. Chen et al. [23] found that addition of 5%wt PSF into epoxy could improve the impact strength while the flexural strength and flexural modulus do not change much. Hu et al. [24] reported that adding PSF into epoxy could significantly improve fracture toughness but might reduce the flexural strength. Other studies have carried out in-depth analyses on the morphology and mechanical properties of PSF/epoxy blends [24,25]. When toughening epoxy resin, different content of PSF form different phase structures which is associated with the toughening effect. Oyanguren et al. [25] studied the phase structure of PSF/epoxy resin materials and found the fracture toughness reached its maximum value of 1.10 MPam^1/2^, which was an improvement of nearly 69.23% when the phase structure of the PSF/DGEBA composites with PSF content of 10 wt% was bi-continuous. Tanaka et al. [26] synthesized polysulphones with cross-linkable pendant vinyl benzyl groups (PSF-VB) as a toughening agent for epoxy resin. Their results indicated that when 10 wt% PSF-VB was added, the fracture toughness of the PSF/epoxy resin composites was increased by 65% while the mechanical properties of composites were not lost. The epoxy resin and PSF-VB were found to be entangled in the presence of dicumyl peroxide (DCP), whereas the phase structure appeared double continuous. However, much effort has been put on the relationship between phase structures and mechanical properties of the cured epoxy resin. Rather, fewer reports stress the influence of PSF on the curing behavior of the epoxy resin, consequently on the change of the phase structure and the mechanical properties after curing.

In this work, PSF resin was used as a toughening agent in the modification of bisphenol A epoxy resin. The effect of PSF on the curing behavior of the epoxy resin at different heating rates was systematically studied and the curing mechanism of the epoxy resin in the presence of PSF was analyzed. The results indicated that the addition of PSF promoted the curing reaction of epoxy resin but does not change its curing mechanism. The toughening of the resultant resin by PSF and the effective improvement in the mechanical properties can be ascribed to the absorption of external energy by different phase separation structures formed by curing induction of the epoxy resin. Relatedly, our studies also revealed that the addition of PSF also improved the heat resistance of the epoxy resin.

## 2. Materials and Methods

### 2.1. Materials

Bisphenol A epoxy resin (E51) with epoxy equivalent 182–192 g/mol, was obtained from Baling Petrochemical Co. Ltd. (Yueyang, China). Polysulfone (PSF) (Udel P1700), with a particle size of ca. 80 μm, from the Acomo Co., Ltd. (Chicago, IL, USA). The curing agent was Dietheyltoluene diamine(DETDA) (Ethacure 100), supplied by the Albemarle Corporation (Carolina, NC, USA). 1, 4-epoxy-butane (THF) was purchased from Sinopharm Chemical Reagent Co., Ltd. (Shanghai, China) and used as solvent.

### 2.2. Preparation of the PSF/Epoxy Composites

The PSF powder was first dissolved in the epoxy resin at 130 °C until the mixed system was completely clarified. Then an appropriate amount of curing agent was added into the mixed system, after which the solution was cooled to 80 °C, followed by mechanical stirring until a uniform mix was obtained. Finally, the mixed system was vacuum defoamed in an oven at 80 °C until there were no more visible bubbles. The mixed system was transferred to a mold coated with release agent and placed in an oven at 120 °C for 4 h. After cooling and demolding, cured product of PSF/epoxy composite were obtained. According to the different weight ratios of PSF to epoxy resin, the samples were recorded as 0, 5, 10, 15 and 20 phr, respectively.

### 2.3. Characterizations

Compared to other methods, involving thermoplastic resins would improve the toughness of the EP without reducing its modulus and heat resistance [12]. Meanwhile, this can also give the epoxy some new properties such as low water absorption and high thermomechanical performances [13], an area that has become a research hotspot in recent years. Rectangular specimens of 36 mm × 13 mm × 3 mm were prepared for DMA analysis, on a single cantilever mode at a frequency of 1 Hz heating from 40 to 250 °C.

The tensile strength and flexural strength/modulus tests were performed following ASTM D638-10 and ASTM D5045-14 Standards. For a typical Flexural strength and modulus test, specimen dimensions were 127 mm × 12.7 mm × 3.2 mm. The fracture toughness of the PSF/epoxy blends was determined by a single-edge notched 3-point flexural test (SENB) according to ASTM D5045-14. Rectangular specimens of 44 mm × 10 mm × 5 mm with a 5 mm length notch were prepared for measurement. A WANCE 203 B-TS micro computer controlled electromechanical universal testing system was applied to perform these measurements. Izod pendulum impact tests were performed based on ASTM D256-10 standard under impact speed and impact energy of 3.5 m/s and 5.5 J, respectively. The sample sizes was 63.5 × 12.7 × 6.35 mm and each sample possessed a constant notch. The fractured surfaces of the cured PSF/epoxy blends with and without etching were analyzed in detail with a Hitachi SU8010 Scanning Electron Microscope (SEM, Tokyo, Japan) system. The surfaces were etched with 1,4-epoxybutane (THF) for 24 h at room temperature to remove the thermoplastic phase. Following that the specimens were dried in vacuum overnight at 100 °C.

The curing kinetics of epoxy resin and epoxy/PSF blends were measured using a NETZSCH 204F1 Differential Scanning Calorimetry (DSC, NETZSCH, Bavarian, Germany). The specimens were heated under a flow of nitrogen (50 mL/min^−1^) from 20 to 350 °C at a heating rate of 5, 10, 15 and 20 °C/min, respectively. The glass transition temperatures (*Tg*) of the PSF/epoxy blends were explored by a TA Q20 differential scanning calorimetry (DSC) instruments from 40 to 250 °C at a heating rate of 10 °C/min under a nitrogen flow (50 mL/min^−1^). Thermogravimetric analyzer (TGA, NETZSCH, Bavarian, Germany) was used to determine the thermal stability of PSF/epoxy. The samples were carried out at 20 °C/min from room temperature up to 900 °C in nitrogen atmosphere.

## 3. Results and Discussion

### 3.1. The Solubility of PSF in Epoxy Resin

**Figure 1:**
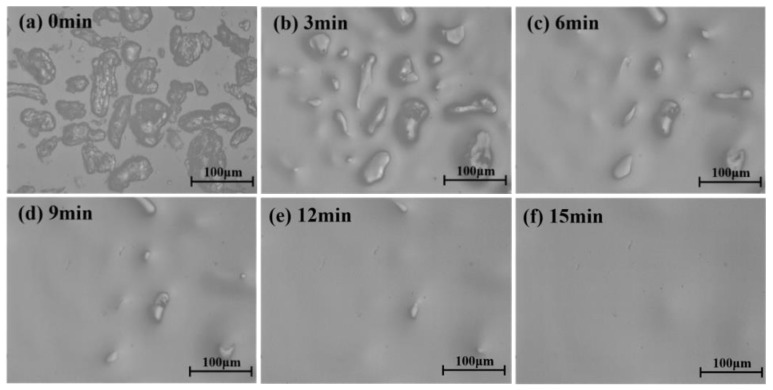
The dissolution process of polysulfone (PSF) powder in epoxy resin at 120 °C, followed by optical microscopy (**a**) 0 min, (**b**) 3 min, (**c**) 6 min, (**d**) 9 min, (**e**) 12 min, (**f**) 15 min.

In order to study the compatibility of epoxy resin and PSF, we observed the dissolution process of PSF powder in epoxy resin and the results are shown in Figure 1. It was found that the PSF was completely dissolved in 15 min at 120 °C. Furthermore, the solution was transparent in general with no visual heterogeneity. These phenomena suggest that PSF has a good solubility in epoxy resin even within a relatively short time.

### 3.2. Curing Kinetics of PSF/Epoxy Resin Blends

**Figure 2:**
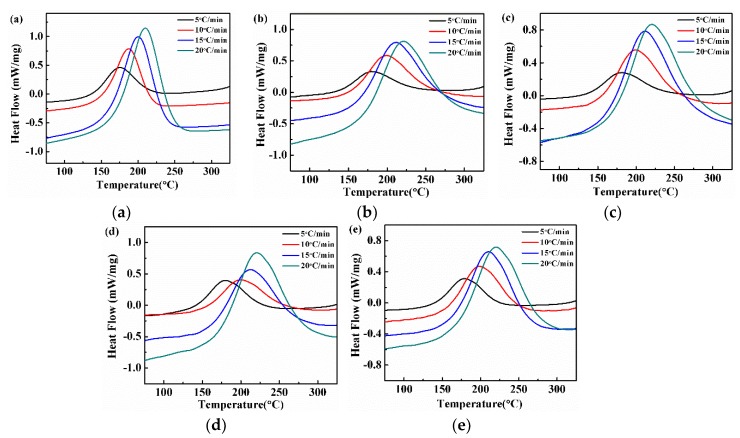
Differential scanning calorimetry (DSC) curves of PSF/epoxy systems at different heating rates of the sample (**a**) 0 phr, (**b**) 5 phr, (**c**) 10 phr, (**d**) 15 phr and (**e**) 20 phr.

It is well known that the curing behaviors of epoxy resin are essentially related to the properties of the cured materials. Thus, the effect of PSF on the curing behaviors of epoxy resin was analyzed by DSC as shown in Figure 2. It is clearly evident that the curing of the epoxy resin is an exothermic process no matter with or without the existence of PSF. As the heating rate in a PSF/epoxy resin system increased, the value of the initial curing temperatures (*T*_i_), the final curing temperature(*T*_f_) and the highest curing temperature rate (*T*_p_) gradually increased. Moreover, the shape of the exothermic peak gradually became sharp. A similar trend was observed for PSF/epoxy systems with different PSF content, which can be ascribed to the steady improvement of thermal inertia, with an increase in the heating rate. In addition, the values of *T*_i_, *T*_f_ and *T*_p_ reduced with the increase in PSF content, which suggests that adding PSF into epoxy resin promotes the curing reaction by reducing the activation energy. This result can be traced to the gradual enhancement of the exothermic lag of the curing reaction on the addition of PSF.

Apparently, the activation energy (Δ*E*) and reaction order (*n*) are crucial kinetic parameters in the curing process and thus the theoretical basis to control the curing process of epoxy resin. Δ*E* directly reflects the degree of difficulty of the curing reaction, which can be calculated according to the Kissinger method [28] as shown in Equation (2). The reaction order (*n*) corresponding to the complexity of the reaction can be used to estimate the curing reaction mechanism. It is quantified according to the Crane equation [29], disclosed in Equation (3).

(1) $$\frac{d(\ln(1)\beta /T_{p}^{2})}{d(1/T_{p})}=-\frac{\Delta E}{R}$$

(2) $$\frac{d(\ln\beta )}{d(1/T_{p})}=-(\frac{\Delta E}{n\;R}{+2T}_{p})$$

where *β* is the heating rate(°C/min), *T*_p_ is the peak temperature, *R* is the ideal gas constant which is 8.314 J/(mol·K), *E* is the activation energy (kJ/mol) and *n* is the reaction order.

**Figure 3:**
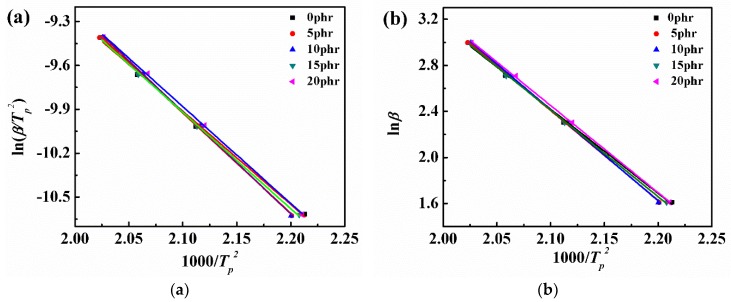
Plots for determining the activation energy of the curing reaction by Kissinger equation (**a**) ln(β/*T*_p_*^2^*) vs. 1000/*T*_p_*,* (**b**) ln*β* vs. 1000/*T*_p_.

According to the Kissinger and the Crane equations, the peak temperature (*T*_p_) of the PSF/epoxy system with different PSF content at different heating rates *β* was substituted into the Equations (2) and (3). After that, the ln(*β/T*_p_*^2^*) and ln*β* were plotted as a function of 1000/*T*_p_, shown in Figure 3a,b, respectively. The slope of each fitted line was obtained by linear regression method. The complex correlation coefficients R^2^ of the two linear fittings all reached 0.99 or higher, indicating an excellent fitting.

Based on the Equations (2) and (3), the calculated values of Δ*E* and *n* are summarized in Table 1. It was found that the value of Δ*E* decreased first and then increased with the growing PSF content, nonetheless, always smaller than that of a pure epoxy resin. Thus, one might speculate that the added PSF promoted the curing reaction of epoxy resin and the promoting effect increased first and then decreased with the increase in PSF content. This might be assocaited with the molecuar structure of polysulfone (both ends have hydroxyl groups) [30], which might have played a catalytic role in the curing reaction of the epoxy resin. At relatively lower PSF content, the amount of hydroxyl groups in the blend system increases with increasing PSF content, thus promoting the curing reaction of epoxy resin. This is respective with the decrease of the apparent activation energy. However, when the PSF content exceeded 15 phr, the viscosity of the whole system increased significantly. Consequently, the steric effects of the PSF macromolecules increase the difficulties of the epoxy resin contacting with the curing agent, to some extent hindering their curing reaction. Moreover, as evident from Table 1, the reaction order (*n*) of each system was stable between 0.87 and 0.88, demonstrating that the addition of PSF does not change the curing reaction mechanism of epoxy resin.

### 3.3. The Chemical Structures of the PSF/Epoxy Resin Blends

**Figure 4:**
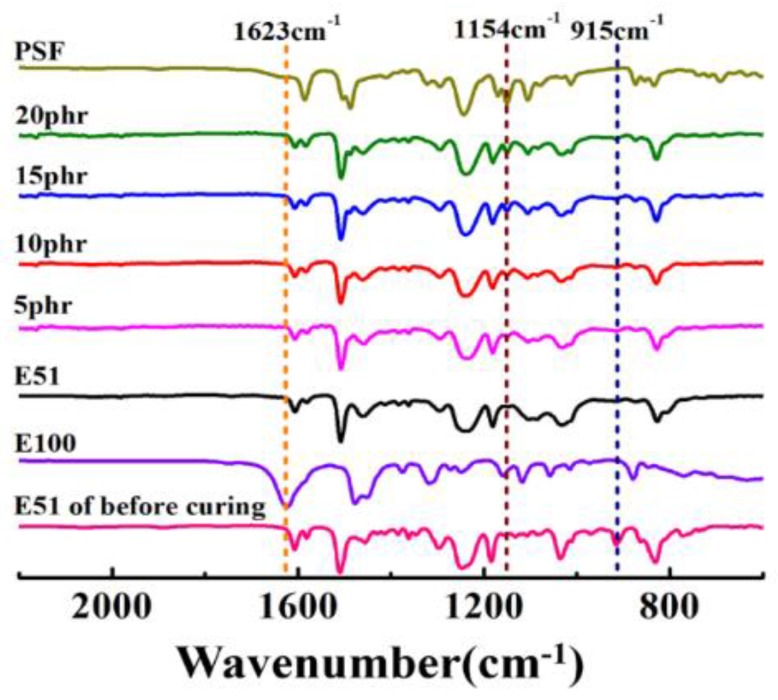
Fourier transform infrared (FT-IR) spectrum of epoxy resin (E51) before curing, the PSF/epoxy blends after curing with different PSF content and PSF.

Various outstanding properties of epoxy resin were obtained by their fully crosslinked structures. Here, in order to elucidate the influence of PSF on the chemical structures of the cured epoxy resin, FT-IR analysis was conducted on the PSF, the neat epoxy resin before curing and the cured PSF/epoxy resin blends as given in Figure 4. The peak of 915 cm^−1^ is the characteristic absorption of epoxy group while the 1623 cm^−1^ is the N–H characteristic peak in the curing agent E100 [27]. Both 915 and 1623 cm^−1^ vanished totally in the PSF/epoxy blends after curing indicating the completion pf the cure reaction. The peak at 1154 cm^−1^ is the stretching vibration peak of the sulfone group [27] and it could be detected after the curing reaction of epoxy resin which means that the addition of PSF would not actively participate the curing reaction of the epoxy resin.

### 3.4. Dynamic Mechanical Thermal Analysis of the PSF/Epoxy Blends

**Figure 5:**
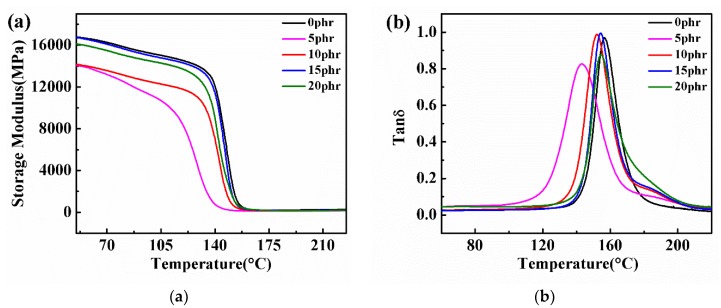
Curves of (**a**) storage modulus *E’* and (**b**) tan *δ* with temperatures of PSF/epoxy cured products containing different PSF content.

The dynamic mechanical thermal analysis gave more insight into their viscoelastic properties as well as the morphologies of PSF/epoxy blends. The variations of the storage modulus of the PSF/epoxy blends after curing with temperatures is shown in Figure 5a. It is found that the growing content of PSF in the blends elevated the storage modulus though it always decreased below that of pure epoxy resin. The tanδ values of the epoxy/PSF blends are plotted against the temperature in Figure 5b. The results present only one peak, implying one *Tg* of the blends. We also notice that the *Tg* of PSF/epoxy blends is smaller than that of pure epoxy resin and it increases with the increase in PSF content. This can be explained in terms of the added PSF reducing the curing degree of the epoxy resin, which results in a decrease in the storage modulus and *Tg* of PSF/epoxy blends [31]. Both the storage modulus and *Tg* of PSF/epoxy blends increasing with the increase in the PSF content may have been caused by the higher storage modulus *Tg* of PSF.

### 3.5. Mechanical Properties of PSF/Epoxy Blends

**Figure 6:**
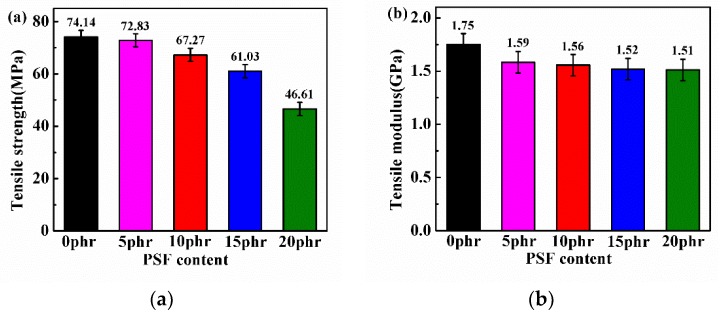
The tensile strength (**a**) and Tensile modulus (**b**) of cured PSF/epoxy blends.

In this work, PSF was also used to improve the mechanical and themal properties of epxoy resin. Therefore, the mechanical propeties including the tensile properties, flexural properties, fracture toughness and impact strength of the cured cured PSF/epoxy blends were illustrated against the varation of PSF content carefully. Figure 6 presents the tensile strength and modulus of cured PSF/epoxy blends. Unfortuately, both the tensile strength and modulus reduced with the addtion of PSF. The tensile strength decreased obviously while the tensile modulus kept a little stable with the the growth of the PSF content. This can be ascribed to the insufficient interfacial adhesion of the polysulfone particles formed by phase separation to the epoxy resin matrix, which results in weaker load transfer capability [32].

**Figure 7:**
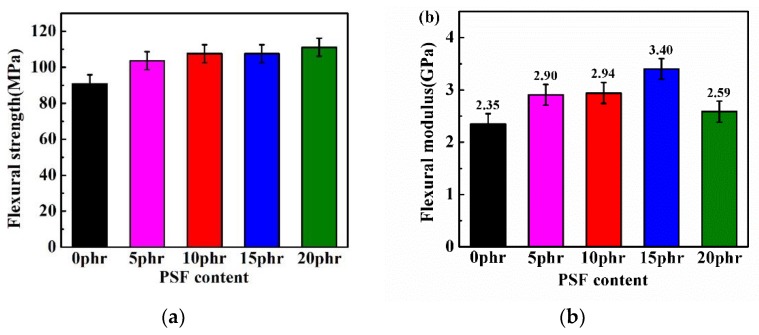
The flexural strength (**a**) and modulus (**b**) of PSF/epoxy blends as a function of PSF content.

The flexural strength and flexural modulus of PSF/epoxy blends is shown in Figure 7. In contrast to the tensile properties, the addition of PSF did not reduce but improved the flexural properties of the epoxy resin. It is clear that both the flexural strength and modulus of the cured PSF/epoxy blends grew slightly with increasing PSF ratio. However, the flexural modulus peaked with 15 phr of PSF while expanding PSF content to 20 phr reduced the flexural modulus although still higher than that of the pure epoxy resin.

The critical stress intensity factor (*K_IC_*) was adopted to inquire into the mechanical interfacial properties or the fracture toughness of the epoxy/PSF blends. The value of *K_IC_* was determined based on the ASTM D5045-14 standard as follows:

(3) $$K_{IC}=\left(\frac{P}{Bw^{1/2}}\right)f\left(x\right)$$

where *P* is the loading weight, *B* is the thickness of the specimen, *W* is the depth(width) of the specimen, *a* is the crack length, *x* is the ratio of the crack length to the depth of specimen, *a/W* and *f(x)* is the calibration factor which is represented as follows:

(4) $$f\left(x\right)=6x^{1/2}\frac{\left[1.99-x\left(1-x\right)\left(2.15-3.93x+2.7x^{2}\right)\right]}{\left(1-2x\right){\left(1-x\right)}^{3/2}}$$

**Figure 8:**
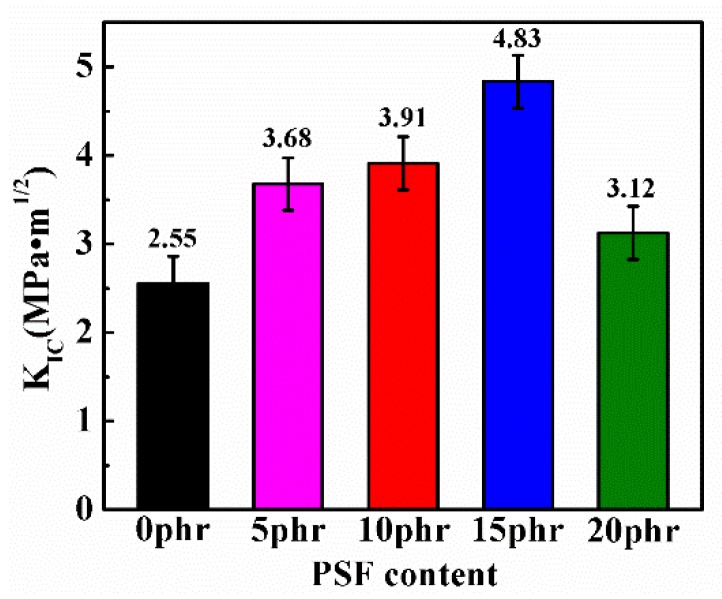
The fracture toughness in terms of critical stress intensity factor (*K_IC_*) of the PSF/epoxy blends as a function of PSF content.

The impact of the PSF content on the fracture toughness in terms of critical stress intensity factor (*K_IC_*) of the PSF/epoxy blends was explored carefully as displayed in Figure 8. Interestingly, all the blend samples showed up an extraodinary improvement of the fracture toughness relative to the neat epoxy resin. Increasing the PSF content is conducive to enhance the fracture toughness while the *K_IC_* of the blend containing 15 phr PSF indicates a maximum fracture toughness. Still increasing the PSF content to 20 phr leads to a slightly smaller *K_IC_* than that of 15 phr PES, nevertheless, still displayed a distinguished enhancement in fracture toughness in comparison with the pure epoxy resin (0 phr).

**Figure 9:**
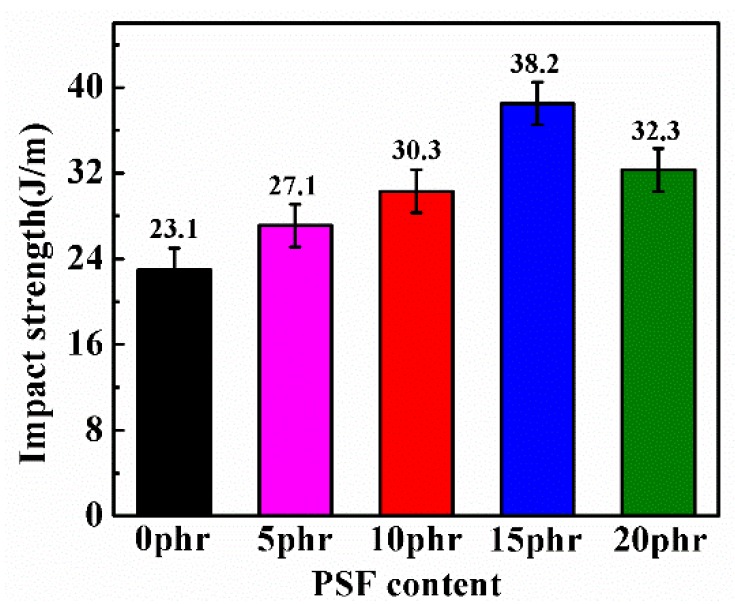
The Impact strength of the PSF/epoxy blends as a function of PSF content.

Pursuing the toughening effect of PSF on epoxy resin further, the Izod pendulum impact tests were conducted on the cured epoxy/PSF blends. As illustrated by Figure 9, a remarkable increase in impact strength was detected for the blends with respect to that of the pure epoxy resin. The best impact strength was obtained at a PSF content of 15 phr with the highest value 38.2 J/m, which was an improvement of ca. 65.3% compared to the neat epoxy resin. When the PSF content reached 20 phr, the impact strength of the PSF/epoxy blends decreased slightly compared to that of the blends containing 20 phr PSF but remained much higher than values of the other samples.

### 3.6. Phase Structures of the PSF/epoxy Blends

**Figure 10:**
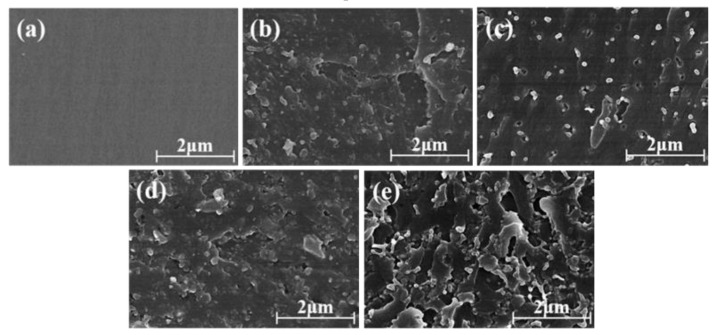
Scanning electron microscope (SEM) images of the fractured surfaces of the PSF/epoxy blends: (**a**) 0 phr, (**b**) 5 phr, (**c**) 10 phr, (**d**)15 phr and (**e**) 20 phr.

In order to understand the relationship between the mechanical propeties and the internal structures, the fractured surfaces of the PSF/epoxy blends obtained from the mechanical tests were observed by SEM as shown in Figure 10. The micrograph of the 0 phr PSF/epoxy blend (Figure 10a) present a smooth fractured surface, demonstrating the brittle property of the pure epoxy resin. In contrast, with the introduction of PSF into the epoxy resin, the morphologies of the fractured surfaces of the blends changed from smooth to rather rough and increasing the PSF content from 5 to 20 phr increased the roughness degree. Additionally, uniform particle extraction was clearly distinguishable from the cross sections of the fractured surface as indicated by Figure 10b. With the increase of PSF content, the particles became more and larger as illustrated by Figure 10c. When the blend consisted of 15 phr PSF and more, crack pinning was easily found besides the particles extraction as evident from Figure 10d,e while the interfaces between the particles and the matrix were well combined. In addition, the polysulfone phases partially contacted with each other and tended to form a continuous phase when the PSF content was increased to 20 phr.

**Figure 11:**
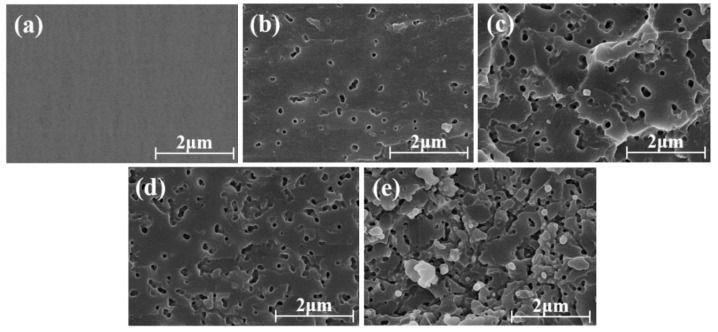
SEM micrographs of the fractured surfaces of PSF/epoxy blends etched with THF; (**a**) 0 phr, (**b**) 5 phr, (**c**) 10 phr, (**d**) 15 phr, (**e**) 20 phr.

PSF can be well dissolved in Tetrahydrofuran (THF). Thus, the PSF/epoxy blends were etched with THF to remove the PSF phase giving more insights into the phase structures of the two components. The resulting morphologies were observed by SEM and displayed in Figure 11. Only a smooth surface appeared in the sample of pure epoxy (0 phr) demonstrating only one epoxy resin phase as indicated by Figure 11a. However, numerous cavities can be distinctly observed in the micrographs of Figure 11b–d which are the spaces occupied by PSF domains before etching. Furthermore, the numbers and sizes of the cavities also grew with the increase in the PSF content. Therefore, the cured PSF/epoxy blends manifested a two-phased morphology in which the PSF domains were uniformly distributed in a continuous epoxy resin matrix. However, when the PSF content was elevated to 20 phr, the cavities were bonded to each other revealing a continuous phase of PSF. Nonetheless, both the continuous and disconnected epoxy particles were surrounded by a continuous phase of PSF, representing a combination of the bicontinuous phase and the phase inversion morphology as depicted by Figure 11e.

The representative phase structures of PSF/epoxy blends could be ascribed to the reaction induced phase separation during curing process of epoxy resin which is related to the content of PSF within the resin system [33]. Before or at the beginning of the curing reaction, the whole resin scheme is generally homogeneous. While the curing reaction proceeds, the molecular weight of the epoxy resin goes up rapidly, leading to the formation of the network structures. With the development of the curing reaction, the compatibility of the PSF resin in the epoxy resin gradually deteriorates and the system is no longer thermodynamically compatible. Consequently, the PSF/epoxy blends resin systems cannot keep stable and a viscoelastic phase-separation process for PSF gradually occurs and evolves. The evolution of the phase separation process of the PSF/epoxy blends is mainly through the spinodal decomposition mechanism controlled by the growing molecular weight of the epoxy resin networks [34]. Depending on the content of the PSF component in the resin system, the phase structure of the resin system may be uniformly dispersed particles, a bicontinuous phase structure or an oppositely rotated structure.

Considering the variations of the mechanical properties of the PSF/epoxy blends, it can be contemplated that the dispersion of the thermoplastic structures within the epoxy resin formed by phase separation plays an important role in the performance of the epoxy resin. The decrease of the tensile strength and modulus with the introduction of the PSF content, arrayed in Figure 6a,b might be the result of the tensile property being mainly influenced by the interaction between the epoxy networks, the crosslinking densities and the defects of the resin [35]. The addition of the PSF in fact reduces the crosslinking densities and also draws some defects to the epoxy resin no matter the kind of phase structure, thus reducing the tensile properties.

In contrast, the uniformly dispersed PSF particles in the epoxy resin matrix as shown in Figure 10b,c exerted a corresponding improvement in the flexural strength and modulus, fracture toughness and the impact strength as depicted in Figure 7, Figure 8 and Figure 9. The flexural property is critically associated with the intensive rigidity of network chains of the resins. With the increase of the PSF content, this intensive rigidity and internal friction of the networks increase, primarily contributed by PSF identical particles [36]. Herein, the flexural strength and modulus of the samples was also elevated. The enhancement of the impact strength and fracture toughness can be attributed to the fact that PSF particles effectively inhibit the crack propagation in the epoxy resin. Types of toughening mechanisms like crack path deflection or plastic deformation of the resin matrix can also be adopted to account for the increase of the toughness of the PSF/epoxy blends [19,37].

As illustrated by Figure 10d, with the formation of a bicontinuous structure, an optimal flexural property, impact strength and fracture toughness can be obtained. The significant enhancement of these mechanical properties is regarded to be associated with the bicontinuous structure of the PSF/epoxy matrix taking advantage of the excellent interfacial adhesion to absorb the crack energy and to shear yield against the crack [38]. Moreover, the crack cannot progress through the continuous PSF phase embedded in the epoxy matrix, resulting in a dominant increase in the toughness of the blends with a bicontinuous phase structure. However, the addition of PSF meanwhile augmented the viscosity of the blended resin, particularly when the PSF content exceeded 15 phr. The matrix of 20 phr PSF corresponding to a phase inversion structure led to lower flexural properties, impact strength and toughness compared to those of the sample containing 15 phr PSF, which might be due to the epoxy resin in the phase inverted structure being discontinuous and lacking a continuous crosslinked network [39].

### 3.7. Glass Transition Temperature of PSF/Epoxy Cured Product

**Figure 12:**
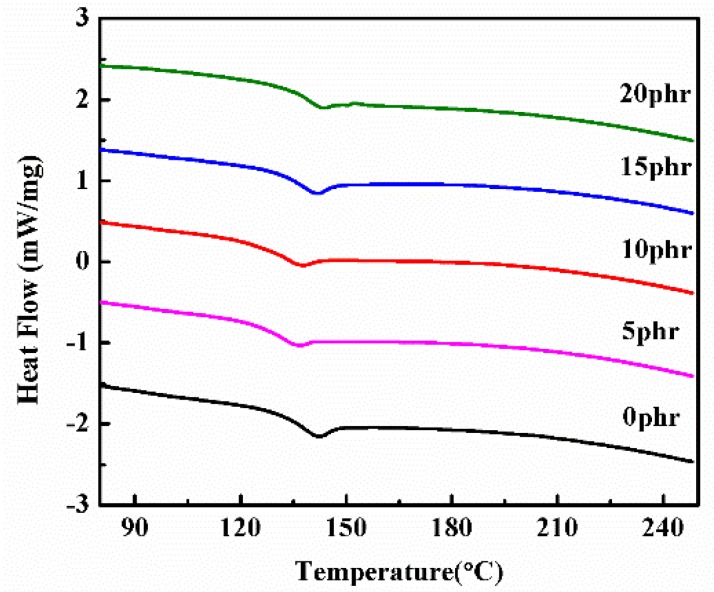
DSC curves of PSF/epoxy cured products with different PSF contents.

In order to study the impact of the PSF on the thermal properties of the epoxy resin, DSC was used to analyze the glass transition temperature (*Tg*) of the PSF/epoxy cured products with different PSF contents. As shown in Figure 12, the addition of PSF did not increase but decreased the *Tg* of the epoxy resin. This might be due to the macromolecular chain of PSF hindering the contact between the epoxy resin and the curing agent, thus lowering the curing degree of the epoxy resin. Meanwhile, the addition of PSF raises the viscosity of the system, which results in an incomplete curing reaction [31,40]. Therefore, the *Tg* of the PSF/epoxy composites was lower than that of the pure epoxy resin. However, when the PSF content increased, the Tg of the PSF/epoxy composites increased as a whole. We attribute this to the fact that polysulfone inherently possesses a higher *Tg*, which could have offset its influence on the curing degree of the epoxy resin. The results of the DSC curve are in good agreement with those from the DMA results.

### 3.8. Thermogravimetric Analysis

**Figure 13:**
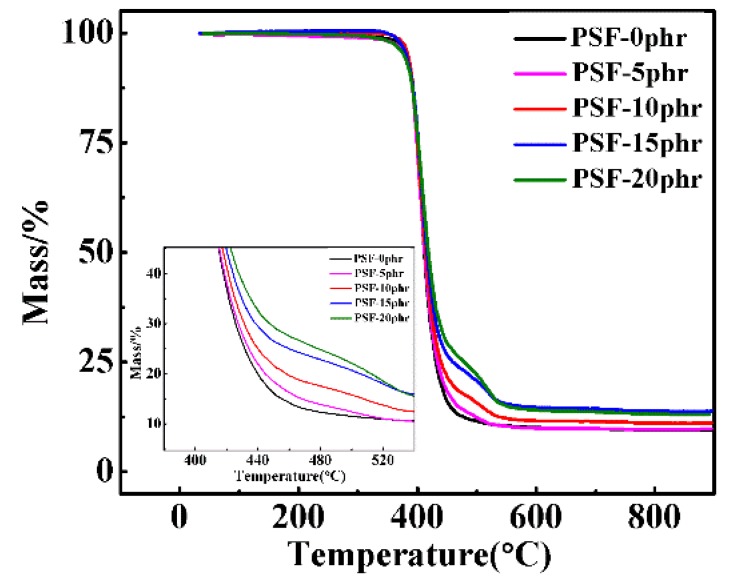
Thermogravimetric curves of PSF/epoxy cured products with different PSF contents.

The thermal stabilities of the cured PSF/epoxy blends were analysed by thermogravimetric analysis and the corresponding thermograms are presented in Figure 13. Two weight loss stage patterns can be clearly distinguished from the TG curves of the PSF/epoxy blends in contrast to the one weight loss stage pattern for pure epoxy resin. The first weight loss is mainly due to the decomposition of the epoxy resin. The second weight loss stage can be ascribed to the inherent high heat resistance properties of the PSF component. The initial and second decomposition temperature of the PSF/epoxy resin increased slightly with the increase of polysulfone content but the change range was not large. We concluded that the addition of PSF did not reduce but effectively enhanced the heat resistance of the epoxy resin system.

**Figure 14:**
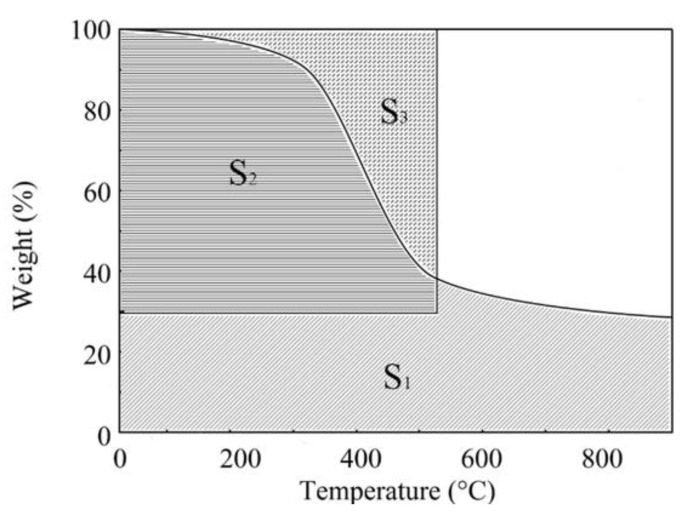
Doyle’s proposition for the calculation of the integrated program decomposition temperature (IPDT).

In order to quantitatively determine the influence of PSF on the thermal stability of epoxy resin, thermal stability parameters such as initial decomposition temperature (IDT) and integrated program decomposition temperature (IPDT) are calculated on the basis of TG curve, as shown in Figure 14. The IPDT was determined from the TG curves according to Dolye’s equation [41]:

IPDT = A*K* (*T*_f_ − *T*_i_) + *T*_i_ (5)

where A* is equal to the integral area, which consists of S1 and S2, divided by the total rectangular plotting area. K* is the ratio of the smaller integral area, S2 to the smaller rectangular area formed by S2 and S3. *T*_i_ is the initial experimental temperature and *T*_f_ is the final experimental temperature. The calculated values of IPDT along with A*K* and other parameters from thermogravimetric analysis are summarized in Table 2. The addition of PSF slightly enhanced the IDT, IPDT, A*K* and *T*_max_ of the blends. This may be due to the high heat resistance of the PSF itself, which improved the heat resistance of the PSF/epoxy resin [42].

## 4. Conclusions

In summary, PSF was employed in this work to improve the mechanical and thermal properties of the epoxy resin. Specifically, the solubility of PSF in epoxy resin and the influence of PSF on the curing process, the chemical structures, the mechanical properties, the phase structures, viscoelastic properties and thermal performance of PSF/epoxy blends were investigated carefully. The dissolution experiment indicated that PSF had good compatibility with the epoxy resin and could be well dissolved into it to prepare the PSF/epoxy blends. The DSC results revealed that the addition of PSF does not upset the curing mechanism but promoted the curing of the epoxy resin. The addition of PSF lowered the reaction enthalpy thus promoted the curing process of the epoxy resin. In fact, PSF did not actively participate in the curing reaction as illustrated by the FT-IR measurements.

The systematic studies of the mechanical properties of the PSF/epoxy blends illuminated that the addition of PSF could significantly enhance the flexural properties, the fracture toughness and impact strength of epoxy resin although it slightly reduced the tensile properties. The improvement of the mechanical properties could be attributed to the representative phase structures resulting from reaction induced phase separation, presenting dispersed particles, bicontinuous and phase inverted structures mostly depending on the PSF content within the blends. The analysis of the results further revealed that the bicontinuous phase morphology obtained during the curing process of the epoxy resin most optimized the mechanical properties. Meanwhile, due to the high heat resistance of PSF components, the thermal stability of the PSF/epoxy blends was also slightly increased compared to that of the pure epoxy resin.

These findings present an interesting benchmark for further understanding the effects, mechanisms and phenomena that surround the phase structure and curing behavior of PSF and epoxy resins. Also, as an example of the modification strategies of epoxy resins, this work reveals that treatment with PSF not only enhances the overall toughness but also maximizes other important properties and tunes the resin for more sophisticated applications.

## Figures and Tables

**Table 1:** Cure kinetic parameters and R2 of PSF/epoxy systems with different PSF content.

Sample	0 phr	5 phr	10 phr	15 phr	20 phr
Δ*E* (kJ/mol)	58.969	57.009	57.997	54.730	55.043
*R*_1_^2^	0.995	0.998	0.998	0.998	0.999
*n*	0.871	0.878	0.88	0.874	0.875
*R*_2_^2^	0.996	0.999	0.999	0.998	0.999

**Table 2:** Thermal stability parameters of polysulfone (PSF)/epoxy blends.

PSF Content	IDT (°C)	IPDT (°C)	*A* ^*^ *K* ^*^	*T*_max_ (°C)
0 phr	386.2	364.6	0.370	412.2
5 phr	389.2	369.3	0.376	412.5
10 phr	392.3	373.5	0.381	414.2
15 phr	394.1	378.1	0.386	416.2
20 phr	395.9	379.4	0.388	418.4
